# Functional Characterization of HLA-G^+^ Regulatory T Cells in HIV-1 Infection

**DOI:** 10.1371/journal.ppat.1003140

**Published:** 2013-01-31

**Authors:** Chun Li, Ilona Toth, Julian Schulze zur Wiesch, Florencia Pereyra, Jennifer Rychert, Eric S. Rosenberg, Jan van Lunzen, Mathias Lichterfeld, Xu G. Yu

**Affiliations:** 1 Ragon Institute of MGH, MIT and Harvard University, Boston, Massachusetts, United States of America; 2 Program of Biological Sciences in Dental Medicine, Harvard University, Cambridge, Massachusetts, United States of America; 3 Department of Internal Medicine, Section of Infectious Diseases, University Medical Center, Hamburg, Germany; 4 Heinrich-Pette-Institute, Leibniz Institute for Experimental Virology, Hamburg, Germany; 5 Infectious Disease Division, Brigham and Women's Hospital, Boston, Massachusetts, United States of America; 6 Infectious Disease Division, Massachusetts General Hospital, Boston, Massachusetts, United States of America; Emory University, United States of America

## Abstract

Regulatory T cells represent a specialized subpopulation of T lymphocytes that may modulate spontaneous HIV-1 disease progression by suppressing immune activation or inhibiting antiviral T cell immune responses. While the effects of classical CD25^hi^ FoxP3^+^ Treg during HIV-1 infection have been analyzed in a series of recent investigations, very little is known about the role of non-classical regulatory T cells that can be phenotypically identified by surface expression of HLA-G or the TGF-β latency-associated peptide (LAP). Here, we show that non-classical HLA-G-expressing CD4 Treg are highly susceptible to HIV-1 infection and significantly reduced in persons with progressive HIV-1 disease courses. Moreover, the proportion of HLA-G^+^ CD4 and CD8 T cells was inversely correlated to markers of HIV-1 associated immune activation. Mechanistically, this corresponded to an increased ability of HLA-G^+^ Treg to reduce bystander immune activation, while only minimally inhibiting the functional properties of HIV-1-specific T cells. Frequencies of LAP^+^ CD4 Treg were not significantly reduced in HIV-1 infection, and unrelated to immune activation. These data indicate an important role of HLA-G^+^ Treg for balancing bystander immune activation and anti-viral immune activity in HIV-1 infection and suggest that the loss of these cells during advanced HIV-1 infection may contribute to immune dysregulation and HIV-1 disease progression.

## Author Summary

HIV-1 causes disease by inducing a chronic inflammatory state that leads to progressive CD4 T cell losses and clinical signs of immune deficiency. Regulatory T cells (Treg) represent a subgroup of T lymphocytes with immunosuppressive activities that can reduce HIV-1 associated immune activation, but may also worsen HIV-1 disease progression by inhibiting T cell responses directed against HIV-1 itself. Here, we describe a non-classical population of regulatory T cells that differ from conventional Treg by the expression of HLA-G, a molecule that contributes to maternal tolerance against semiallogeneic fetal tissue during pregnancy. We show that HLA-G-expressing Treg have a unique functional ability to reduce harmful bystander immune activation, while minimally inhibiting potentially beneficial T cell-mediated immune responses against HIV-1. In this way, HLA-G-expressing Treg may represent a previously unrecognized barrier against HIV-1 associated immune activation and a possible target for future immunotherapeutic interventions in HIV-1 infection.

## Introduction

The hallmark of HIV-1 infection is a progressive reduction of CD4 T cells. The main function of these cells is to provide antigen-specific helper cell activity against a wide panel of microbial antigens, however, some of these cells also have regulatory immunosuppressive activities. Classical regulatory T cells (Treg) are immunophenotypically defined as being CD25^hi^ and CD127^lo^, and they intracellularly express the Forkhead Box P3 protein (FoxP3) [1]. The importance of classical Treg for maintaining immune homeostasis has been highlighted by signs of autoimmune pathology that occur in the setting of deficient Treg activity [2], [3]. During progressive HIV-1 infection, the relative frequency of classical Treg is increased, while their absolute counts are reduced as a consequence of lower total CD4 T cell counts [4]. This indicates that classical Treg decline at a slower rate than conventional CD4 T cells during progressive HIV-1 infection, and suggests that these cells may play an important role in the immune pathogenesis of HIV-1 infection. Functional data from previous studies indeed demonstrated that classical Treg can potently suppress HIV-1-specific T cell responses [5], [6], and in this way may contribute to the failure of achieving T cell-mediated immune control of HIV-1 replication. However, classical Treg may also have beneficial effects on HIV-1 disease progression by reducing the deleterious consequences of HIV-1 associated immune activation [7], [8].

Recently, several alternative Treg populations have been identified that differ from classical Treg by the lack of intracellular FoxP3 expression. One group of such non-classical Tregs is defined by surface expression of HLA-G [9], an HLA class Ib molecule that is mainly expressed on placental trophoblasts. However, ectopic expression of HLA-G can also be observed on small populations of peripheral blood CD4 and CD8 T cells, which seem to be enriched at sites of inflammation [9]. These cells have the ability to suppress proliferation of T lymphocytes in a cell-contact independent manner, and their regulatory effects are reversible following neutralization with HLA-G blocking antibodies [10]. Previous reports suggested that the proportion of HLA-G-expressing CD8 T lymphocytes is increased during HIV-1 infection [11], however, such investigations were conducted in unselected populations of HIV-1 positive persons, and did not address the functional role of HLA-G^+^ T cells during different stages of HIV-1 disease progression.

A second group of non-classical Tregs is characterized by surface expression of the latency-associated peptide (LAP), a membrane bound form of TGF-β [12]. These LAP^+^ CD4 T cells lack FoxP3 expression but can inhibit proliferative activities of T lymphocytes *in vitro* and *in vivo*. Under physiologic conditions, a small proportion of LAP-expressing CD4 T cells can be detected in human peripheral blood [12]. The numeric distribution and functional role of LAP^+^ CD4 Treg during HIV-1 infection is not known.

In the present study, we systematically analyzed the expression and function of HLA-G-and LAP-expressing Tregs in patients with different stages of HIV-1 disease infection. Our results indicate a profound reduction of HLA-G^+^ CD4 Treg in individuals with progressive HIV-1 disease that may stem from a higher susceptibility of these cells to HIV-1 infection, and functionally contribute to HIV-1-associated immune overactivation.

## Methods

### Study participants

HIV-infected patients and HIV-1 seronegative control persons were recruited according to protocols approved by the Institutional Review Board of the Massachusetts General Hospital in Boston. Samples of mononuclear cells extracted from lymph nodes and peripheral blood were obtained from HIV-1 infected study patients recruited at the University of Hamburg (Germany) according to a protocol approved by the local Ethics Committee.

### Ethics statement

All subjects gave written informed consent and the study was approved by the Institutional Review Board of Massachusetts General Hospital/Partners Healthcare.

### Immunophenotypic analysis

Peripheral blood mononuclear cells (PBMC) were isolated from whole blood using Ficoll density centrifugation. Lymph node mononuclear cells (LNMC) were extracted from freshly-excised lymph node samples according to routine procedures. PBMC or LNMC were stained with LIVE/DEAD cell viability dye (Invitrogen, Carlsbad, CA) and monoclonal antibodies directed against CD4, CD25, CD127, CD45RA, CCR7 (BD Biosciences, San Jose, CA), CD57 and PD-1 (Biolegend, San Diego, CA), CD8 (Invitrogen), HLA-G (clone MEM-G/9, Abcam, Cambridge, MA), LAP (clone 27232, R&D systems, Minneapolis, MN) and, when indicated, LILRB1 (clone HP-F1, ebioscience, San Diego, CA). After incubation for 20 minutes at room temperature, cells were fixed with PBS containing 0.5% fetal calf serum and 1% formaldehyde. Anti-FoxP3 antibodies (ebioscience) were used with a dedicated staining buffer (ebioscience) per the manufacturer's instruction. Subsequently, cells were acquired on an LSR II flow cytometer (BD Biosciences, San Jose, CA) using FACSDiva software. Data were analyzed using FlowJo software (Tree Star, Ashland, OR).

### Cell isolation and sorting

Indicated total CD4 or CD8 T cell populations were isolated using a negative cell purification kit (StemCell Technologies, BC, Canada), according to the manufacturer's instructions. Cell purity was >90% in all cases. Classical LAP^−^ HLA-G^−^ CD25^hi^ CD4 T cells, HLA-G^+^ CD4 T cells, LAP^+^ CD4 T cells and a control cell population of LAP^−^ HLA-G^−^ CD25^−^ CD4 T cells were sorted on a FACSAria instrument (BD Biosciences) at 70 pounds per square inch. For isolation of CD8 Treg subsets, purified bulk CD8 T cells were sorted into three T cell subsets: HLA-G^+^ CD8 T cells, CD25^hi^ CD28^−^ CD8 T cells and a control cell population of HLA-G^−^ CD25^−^ CD8 T cells, using similar sorting conditions.

### Proliferation assay

PBMC from HIV-1 infected individuals were stained with 0.25 µM carboxyfluorescein succinimidyl ester (CFSE; Invitrogen) and mixed with sorted autologous Treg populations or control T cells without regulatory activity at a ratio of 4∶1. Afterwards, cells were stimulated with a pool of overlapping peptides spanning the clade B consensus sequence of HIV-1 gag, a pool of overlapping peptides spanning the entire sequence of human CMV pp65 (concentration of 2 µg/ml per peptide), or PHA. After incubation for 6 days, cells were washed, stained with viability dye and surface antibodies, fixed and acquired on an LSR II flow cytometer. Suppression of T cell proliferation by Tregs was calculated as: (T cell proliferation (%) in the non-Treg co-culture – T cell proliferation (%) in the Treg co-culture)/T cell proliferation (%) in the non-Treg co-culture.

### Intracellular cytokine staining

CFSE-stained responder T cells from HIV-1-infected patients were mixed with sorted autologous Treg populations or control CD4 T cells at a ratio of 2∶1. Cells were then stimulated with a pool of overlapping peptides spanning HIV-1 gag (concentration of 2 µg/ml per peptide) in the presence of antibodies directed against CD28 and CD49d (2 µg/ml). Cells were incubated for 6 h at 37°C, and Brefeldin A was added at 5 µg/ml after the first hour of incubation. Afterwards, cells were stained with viability dye and surface antibodies, fixed, permeabilized using a commercial kit (Caltag, Burlingame, CA), and subjected to intracellular cytokine staining with monoclonal antibodies against interferon-γ and IL-2 (BD Biosciences). Following final washes, cells were acquired on an LSR II instrument.

### Assessment of bystander activation

Responder T cells from healthy individuals were mixed with sorted autologous Treg populations or autologous control T cells without regulatory activities at a ratio of 2∶1. Following stimulation of cells with Staphylococcal Enterotoxin B (SEB, 5 µg/ml, kindly provided by Dr. Eric J. Sundberg, University of Maryland), cells were incubated at 37°C for 4 days. Afterwards, cells were stained with antibodies against CD4, CD8, CD38, HLA-DR, CD69 and Vβ13.1 and viability dye before being subjected to flow cytometric acquisition on an LSR II instrument. The surface expression of activation markers in responder T cells was analyzed after gating on T cells. Treg-dependent suppression of bystander activation was calculated as: (CD38/HLA-DR/CD69-expressing T cells (%) in the non-Treg co-culture – CD38/HLA-DR/CD69-expressing T cells (%) in the Treg co-culture)/CD38/HLA-DR/CD69-expressing T cells (%) in the non-Treg co-culture.

### Detection of HLA-G by western blot

HLA-G^+^ and HLA-G^−^ CD3 T cells were isolated by immunomagnetic enrichment and cultured in IL-2 supplemented medium for 4 days. Equal amounts of culture supernatants and cell lysates were then subjected to SDS-PAGE (8 to 16% Tris-glycine gels, Invitrogen), electroblotted and incubated with HLA-G antibodies (clone 4H84, Abcam), followed by visualization with horseradish peroxidase (HRP)-labeled secondary antibodies and enhanced chemiluminescence (ECL) detection reactions (GE Healthcare, Little Chalfont, UK) according to standard protocols [13].

### *Ex-vivo* infection assays

CD4 T cells were activated with recombinant IL-2 (50 U/ml) and an anti-CD3/CD8 bi-specific antibody (0.5 µg/ml). On day 5, cells were infected with GFP-encoding X4- (NL4-3, MOI = 0.02) or R5- (Ba-L, MOI = 0.07) tropic viral strains [14] (kindly provided by Dr. Dan Littman, New York University) for 4 h, or with a YFP-encoding VSV-G-pseudotyped HIV-1 vector (MOI = 0.02) (kindly provided by Dr. Abraham Brass, University of Massachusetts) for 2 h at 37°C. After two washes, cells were plated at 5×10^5^ cells per well in a 24-well plate. On day 2 (VSV-G-pseudotyped virus) or day 4 (X4-/R5-tropic viruses), cells were stained with surface antibodies and viability dye and analyzed on an LSR II instrument. For infection of quiescent cells, negatively-selected CD4 T cells with a purity of >95% were directly infected with the described HIV-1 constructs. After *in vitro* culture for 96 h in the absence of exogenous IL-2, cells were analyzed by flow cytometry.

### Statistical analysis

Data are expressed as mean and standard deviation/standard error, or as box and whisker plots indicating the median, the 25% and 75% percentile and the minimum and maximum of all data. Differences between different cohorts or different experimental conditions were tested for statistical significance using Mann-Whitney U test, paired T test or one-way ANOVA, followed by post-hoc analysis using Tukey's multiple comparison test, as appropriate. Spearman correlation was used to assess the association between two variables. A p-value of 0.05 was considered significant. The level of significance was labeled as: *:p<0.05; **:p<0.01; ***:p<0.001.

## Results

### Reduced frequency of HLA-G^+^ Treg in progressive HIV-1 infection

Investigations of T cells with regulatory properties in HIV-1 infection have so far been mostly limited to classical, CD25^hi^ and/or FoxP3 expressing Treg. To analyze the role of alternative, non-classical Treg populations in patients infected with HIV-1, we initially focused on the recently described population of Treg defined by surface expression of HLA-G [9]. These cells do not express FoxP3 or CD25 (Figure S1), and are phenotypically and functionally distinct from classical Treg [9], [10]. To analyze these cells in HIV-1 infection, we used flow cytometry to determine the relative and absolute numbers of HLA-G^+^ CD4 and CD8 T cells in treatment-naïve HIV-1 infected individuals with chronic progressive infection (n = 28, median viral load: 48,215 copies/ml [IQR 20,187–685,000]; median CD4 cell count: 396/µl [IQR 204–652]), spontaneous control of HIV-1 replication (n = 24, viral load <1000 copies/ml; median CD4 cell count: 924/µl [IQR 347–1879]), or patients with primary HIV-1 infection and seroconversion within 3 months prior to recruitment (n = 22, median viral load: 99,900 copies/ml [IQR 36,600–2,790,000]; median CD4 cell count: 475/µl [IQR 265–1047]). HIV-1 infected persons successfully treated with Highly Active Antiretroviral Therapy (HAART) (n = 26, viral load <50 copies/ml; median CD4 cell count: 402/µl [IQR 242–1493]), as well as a cohort of HIV-1 negative persons (n = 21), were recruited for control purposes.

**Figure 1:**
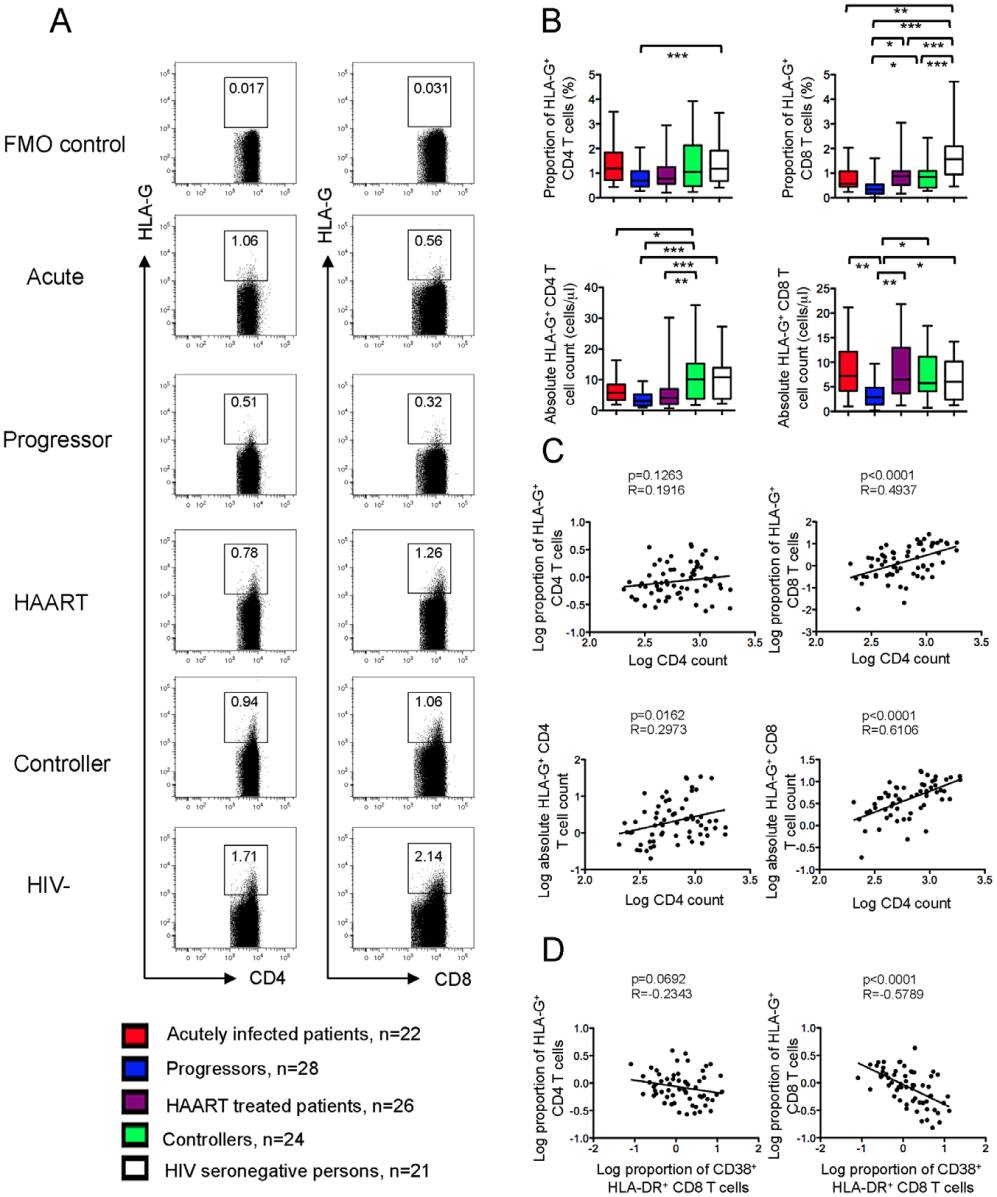
Diminished proportions of HLA-G^+^ CD4 and CD8 T cells in progressive HIV-1 infection. (A): Representative flow cytometry dot plots reflecting the proportions of HLA-G^+^ CD4 and CD8 T cells in indicated study subjects. FMO control reflects “fluorescence minus one” control condition without addition of HLA-G antibodies. (B): Box and Whisker plots summarizing the relative proportions and absolute numbers of HLA-G^+^ CD4 and CD8 T cells in indicated study cohorts. ANOVA followed by post-hoc analysis with Tukey's Multiple Comparison Test was used to determine significance. (C): Correlations between frequencies of HLA-G^+^ T cells and total CD4 T cell counts in controllers (n = 23), progressors (n = 27) and HIV seronegative individuals (n = 15). (D): Correlations between proportions of HLA-G^+^ Treg and CD8 T cell immune activation determined by surface expression of CD38 and HLA-DR in controllers (n = 19), progressors (n = 20), and HIV seronegative individuals (n = 15). (C/D): Spearman's correlation coefficient is shown.

Consistent with prior reports [15], we observed that relative proportions of classical CD25^hi^ CD127^lo^ CD4 Treg were increased in progressive HIV-1 infection, while absolute Treg numbers were decreased (Figure S2); no correlation was found between relative proportions of classical Treg and levels of immune activation (Figure S2). In contrast, we observed that the relative and absolute numbers of HLA-G-expressing CD4 T cells were lowest in HIV-1 progressors, while no significant difference was found between the numbers of HLA-G^+^ CD4 T cells in any of the other HIV-1 patient cohorts and HIV-1 negative persons (Figure 1 A/B). The relative frequencies of HLA-G^+^ CD8 T cells were lower in all HIV-1 infected patient populations compared to HIV-1 negative persons; this reduction was again most pronounced in persons with untreated progressive disease. Notably, the numbers of HLA-G-expressing CD4 and CD8 T cells were positively correlated to total CD4 T cell counts (Figure 1C), and proportions of HLA-G^+^ T cells were inversely associated with corresponding levels of immune activation on T cells, as determined by surface expression of HLA-DR and CD38 (Figure 1D). These data indicate a selective numerical decrease of HLA-G-expressing T cells in chronic progressive HIV-1 infection, and suggest that a reduction of HLA-G^+^ Treg may contribute to higher levels of immune activation during progressive HIV-1 infection.

**Figure 2:**
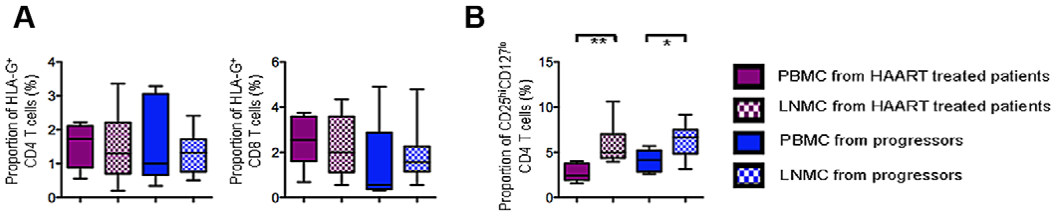
Analysis of HLA-G^+^ Treg in lymph nodes and peripheral blood during HIV-1 infection. (A) Proportions of HLA-G^+^ CD4 and CD8 Treg in lymph node and peripheral blood samples. (B) Corresponding analysis of the frequencies of classical CD25^hi^ CD127^lo^ CD4 Treg in lymph node and peripheral blood samples. Box and Whisker plots represent cumulative data from n = 5 PBMC/n = 9 LNMC from HAART-treated HIV-1 patients and n = 5 PBMC/n = 12 LNMC from untreated HIV-1 patients. Significance between groups was tested by Mann Whitney U test.

Since HLA-G^+^ Treg express multiple tissue homing factors [16], a redistribution of these cells to lymphoid tissues may be responsible for the apparent reduction of HLA-G-expressing Treg in the peripheral blood during progressive HIV-1 infection. To investigate this, we analyzed the proportion of HLA-G^+^ T cells in lymph node and peripheral blood samples collected from patients treated with antiretroviral therapy (HIV-1 viral load<75 copies/ml, median CD4 count: 762/µl [IQR 528–1,152]) or with untreated progressive HIV-1 infection (median HIV-1 viral load: 73,500 copies/ml [IQR 1,300–252,000], median CD4 count: 430/µl [IQR 254–1,267]). Within these patients, proportions of HLA-G^+^ CD4 and CD8 Treg in lymph nodes and peripheral blood were not significantly different, suggesting that compartmentalization of HLA-G^+^ Treg to lymph nodes does not represent the major reason explaining the decreased number of circulating HLA-G^+^ Treg in progressive HIV-1 infection (Figure 2A). In contrast, classical CD25^hi^ CD127^lo^ Treg were significantly enriched in lymph nodes compared to peripheral blood in patients on and off HAART, consistent with previous results [17] (Figure 2B).

We next investigated whether the reduced frequencies of circulating HLA-G^+^ Treg during progressive HIV-1 infection are associated with an altered phenotypic differentiation or maturation status. We found that in all study cohorts, the T cell subset distribution of HLA-G^+^ CD4 T cells into naïve, central-memory, effector-memory and terminally-differentiated CD4 T cells was not substantially different from corresponding bulk CD4 T cells (Figure S3). Moreover, the expression of CD57 and PD-1, two surface markers associated with senescence and exhaustion of T cells, was not markedly different between HLA-G^+^ CD4 T cells and the respective bulk CD4 T cells (Figure S4). In contrast, we noted that in all study cohorts, HLA-G^+^ CD8 T cells tended to have a more immature naïve or central-memory phenotype when compared to reference bulk CD8 cell populations (Figure S3). There was also a trend for reduced surface expression of CD57 surface expression on HLA-G^+^ CD8 T cells in comparison to corresponding bulk CD8 T cells (Figure S4). Overall, these data indicate that during HIV-1 infection, HLA-G-expressing CD8, but not CD4 T cells, are skewed to a more immature differentiation status, but this difference is not correlated to the rates of spontaneous HIV-1 disease progression.

### Frequencies of LAP^+^ Treg in HIV-1 infection

T cells expressing LAP, a membrane-bound form of TGF-β, have recently been characterized as an alternative, FoxP3-negative population of lymphocytes with immunosuppressive properties [12] [18]. To determine whether this non-classical population of regulatory cells is involved in HIV-1 disease pathogenesis, we analyzed the frequency of LAP^+^ T cells in our study cohorts. We did not observe significant differences in the proportions of LAP^+^ T cells between our study groups (Figure S5). Absolute numbers of LAP^+^ CD4 Treg were positively associated with total CD4 T cell counts (Figure S5), and were lowest in progressors, likely reflecting the decline of total CD4 T cells in this patient population (Figure S5). Proportions of neither LAP^+^ CD4 nor LAP^+^ CD8 T cells were significantly associated with corresponding levels of immune activation (Figure S5). LAP^+^ T cells did not substantially differ from bulk T cells in terms of T cell subset distribution, although LAP^+^ CD8 T cells appeared to be slightly overrepresented in central-memory cells during HIV-1 infection (Figure S6). No difference was found between the surface expression of PD-1 and CD57 on LAP^+^ T cells and bulk T cells (Figure S4). Taken together, these results do not suggest that LAP^+^ T cells play a major role in HIV-1 immune protection or restriction of HIV-1 associated immune activation.

### HLA-G^+^ Treg minimally inhibit functional properties of HIV-1-specific T cells

**Figure 3:**
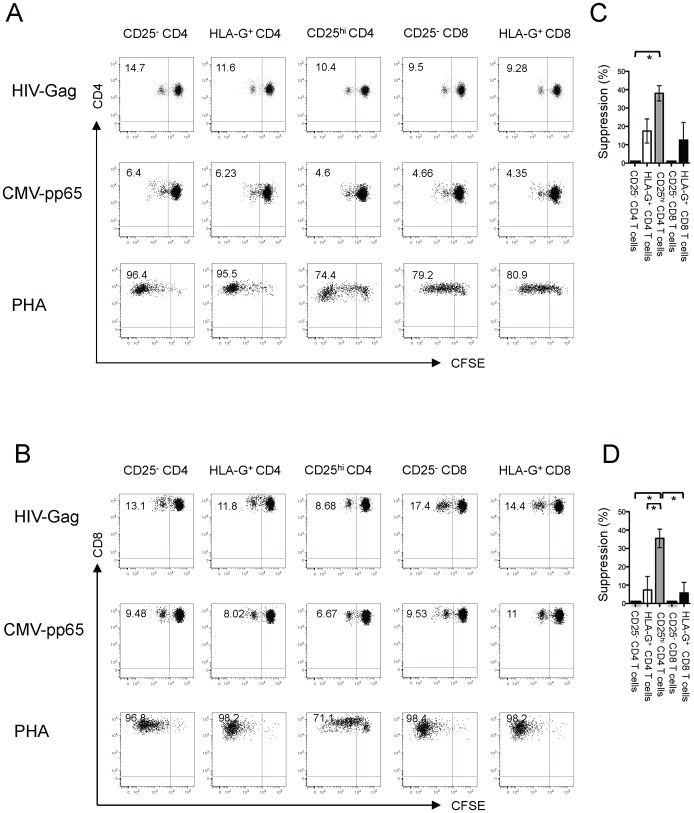
HLA-G^+^ Treg minimally inhibit proliferative activities of antigen-specific T cells. (A–B): Representative dot plots reflecting proliferative activities of HIV-1-, CMV-, or PHA-stimulated CD4 (A) or CD8 (B) T cells from HIV controllers following incubation with indicated autologous Treg subsets or HLA-G^−^ CD25^−^ control cells. (C–D): Cumulative data reflecting the Treg-mediated suppression of HIV-1-specific CD4 (C) or CD8 (D) T cell proliferation from n = 3 HIV-1 controllers. Significance was tested by paired T test.

A functional hallmark of classical Treg is their ability to inhibit antigen-specific T cell responses [19]. Prior work has shown that non-classical Tregs can also inhibit proliferative properties of T cells, but their functional effects on HIV-1-specific T cells remain unclear [9]. To investigate this, CFSE-labeled PBMC from HIV-1 controllers were stimulated with viral peptides or PHA and individually mixed with sorted autologous HLA-G^+^ CD4 Treg, HLA-G^+^ CD8 Treg or classical CD25^hi^ CD4 Treg; HLA-G^−^ CD25^−^ CD4 or CD8 T cells were added as negative controls. Subsequently, proliferation of HIV- and CMV-specific T cells was monitored after six days of culture. These experiments demonstrated suppressive effects of classical CD25^hi^ Treg on the proliferative activities of HIV-1- and CMV-specific CD4 and CD8 T cells, consistent with prior reports showing potent Treg-mediated inhibition of T cell proliferation [5]. In contrast, HLA-G^+^ Treg did not effectively suppress the proliferative activity of autologous virus-specific CD4 (Figure 3A/C) or CD8 (Figure 3B/D) T cells in these study patients. LAP-expressing CD4 Treg had a moderate suppressive effect on proliferative activities of HIV-1-specific T cells (Figure S7). None of the tested classical or non-classical Treg populations had a measurable impact on interferon-γ or IL-2 secretion in HIV-1-specific CD8 T cells (Figure S8). Taken together, these data show that HLA-G^+^ Treg have minimal effects on the functional activities of virus-specific T cell responses in controllers.

### HLA-G-expressing Treg selectively reduce bystander activation

To further explore the role of non-classical Tregs in HIV-1 disease pathogenesis, we focused on how these cells influence T cell activation. Activation of T lymphocytes can either occur through direct antigenic triggering of the TCR, or by mechanisms involving a TCR-independent mode of T cell stimulation, commonly referred to as “bystander activation” [20], [21]. Both of these pathways seem to contribute to the pathological immune activation observed during progressive HIV-1 infection [22], [23], and may be influenced by the non-classical Treg populations described in this manuscript. As a functional assay to investigate and quantify the effects of non-classical Tregs on TCR-dependent and bystander immune activation, we stimulated T cells with Staphylococcal Enterotoxin B (SEB), an antigen that elicits T cell responses by a broad panel of different TCR clonotypes, but cannot be recognized by T cells using TCR Vβ13.1 [24], [25]. Immune activation in Vβ13.1-expressing T cells following exposure to SEB can therefore only be attributed to bystander activation, while immune activation in Vβ13.1-negative T cells after SEB exposure reflects classical TCR-dependent activation.

**Figure 4:**
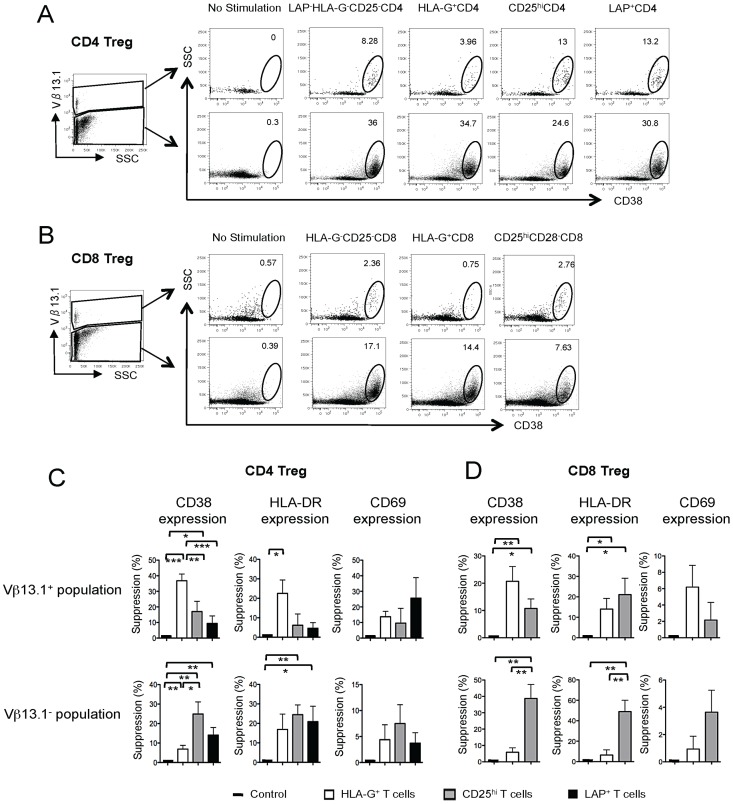
HLA-G^+^ Treg reduce bystander activation of T cells. (A–B): Representative flow cytometry dot plots reflecting the surface expression of CD38 on Vβ13.1^+^ and Vβ13.1^−^ responder T cells following exposure to indicated CD4 (A) or CD8 (B) Treg cell populations or negative control CD4 or CD8 T cells from HIV-seronegative donors. (C–D): Cumulative data representing relative suppression of CD38, HLA-DR and CD69 on Vβ13.1^+^ and Vβ13.1^−^ responder T cells following exposure to indicated CD4 (C) or CD8 (D) Treg cell populations. Mean and standard deviation from n = 8 HIV-1 negative study subjects are shown. Significance was tested by paired T test.

To analyze the effects of non-classical Tregs on immune activation, SEB-stimulated responder T cells were individually co-cultured with autologous populations of sorted LAP^+^ CD4 Treg, HLA-G^+^ CD4 Treg, or classical LAP^−^ HLA-G^−^ CD25^hi^ CD4 Treg; LAP^−^ HLA-G^−^ CD25^−^ CD4 T cells were added for control purposes. Alternatively, HLA-G^+^ CD8 T cells, CD25^hi^ CD28^−^ CD8 T cells or HLA-G^−^ CD25^−^ CD8 control cells were added to autologous SEB-stimulated responder T cells. On day 4 of culture, immune activation was measured by flow cytometric analysis of CD38, HLA-DR and CD69 surface expression in Vβ13.1-expressing and Vβ13.1-negative T cells. As demonstrated in Figure 4, we observed that classical CD25^hi^ Treg potently suppressed CD38/HLA-DR expression in Vβ13.1-negative T cells, consistent with prior reports about the immunosuppressive properties these cells [5]. In contrast, HLA-G-expressing Treg led to a significantly reduced surface expression of CD38 on Vβ13.1-expressing T cells, but had limited effects on immune activation of Vβ13.1-negative cells. This selective inhibitory effect on bystander activation was seen both for HLA-G^+^ CD4 (Figure 4A/C) and CD8 (Figure 4B/D) T cells and substantially exceeded regulatory effects on bystander activation of classical CD25^hi^ Treg or LAP^+^ Treg. None of the tested Treg populations significantly affected CD69 expression on responder cells over the 4-day incubation period, likely because in comparison to CD38, CD69 is only transiently upregulated for a short period after immune activation [26], and therefore could not be properly evaluated in our 4-day co-culture experiment.

**Figure 5:**
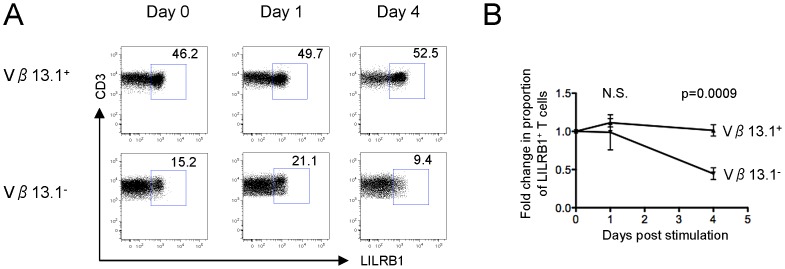
Stable LILRB1 expression on T cells after “bystander activation”. (A) Representative dot plots demonstrating proportions of LILRB1-expressing Vβ13.1^+^ and Vβ13.1^−^ T cells after activation with SEB over indicated time course. (B): Fold change of LILRB1 expression on Vβ13.1^+^ or Vβ13.1^−^ T cells at indicated time points. Mean and standard error from 7 different HIV-1 negative donors are shown. Significance was tested by paired T test.

To explore reasons for the differential susceptibility of Vβ13.1-positive and Vβ13.1-negative responder T cells to classical and non-classical Tregs, we analyzed the dynamics of LILRB1 surface expression on responder T cells over a 4-day incubation period. LILRB1 can effectively inhibit functional properties of T cells [27] and represents one of the highest-affinity receptors for HLA-G [28], which is secreted by HLA-G^+^ Treg (Figure S9) and responsible for the immunomodulatory effects of HLA-G^+^ Treg [9], [10]. Interestingly, we observed that following TCR-dependent T cell activation, LILRB1 surface expression on responder T cells declined, while stable or slightly increased LILRB1 surface expression was observed on Vβ13.1-negative T cell after “bystander activation” (Figure 5). Overall, these data indicate that HLA-G^+^ Treg differ from alternative Treg populations by their ability to reduce bystander activation of T cells, and suggest that TCR-dependent and TCR-independent mechanisms of immune activation are associated with altered susceptibilities to inhibitory effects of classical and non-classical Tregs.

### HLA-G-expressing CD4 T cells are highly susceptible to HIV-1 infection

**Figure 6:**
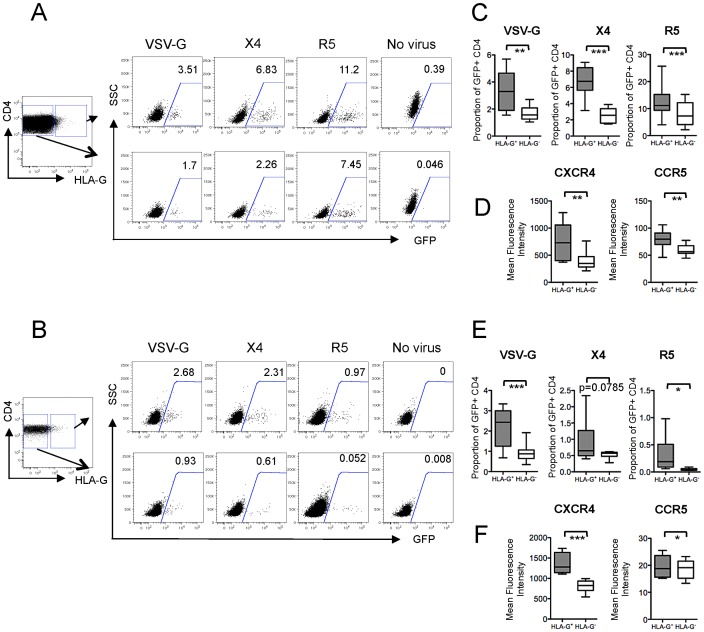
Susceptibility of HLA-G^+^ Treg to HIV-1 infection. (A–B) Representative flow cytometry dot plots reflecting the proportions of GFP-expressing HLA-G^+^ or HLA-G^−^ CD4 T cells after infection with GFP-encoding X4- or R5-tropic HIV-1 viruses, or a VSV-G-pseudotyped HIV-1 vector. Cells from HIV-1 seronegative donors were infected after *in vitro* activation (A) or directly after *ex-vivo* isolation (B). (C/E) Box and Whisker plots reflecting the proportions of GFP-positive CD4 T cells after infection with indicated viral strains, with (C) or without prior *in vitro* activation (E). (D/F): Expression of HIV-1 co-receptors CXCR4 and CCR5 on HLA-G^+^ or HLA-G^−^ cells, with (D) or without prior *ex-vivo* activation (F). (C–F): Data from n = 10 CD4 T cell populations with prior *in vitro* activation and n = 9 CD4 T cell populations with direct *ex-vivo* infection are shown. Significance was tested by paired T test.

Conventional CD25^hi^ CD4 Treg express HIV-1 co-receptors and are targets for HIV-1 infection [29], [30]. Direct HIV-1 infection of HLA-G^+^ CD4 Treg may contribute to the reduction of these cells in progressive HIV-1 infection. To investigate this, we analyzed the susceptibility of HLA-G^+^ CD4 Treg to X4- or R5-tropic HIV-1 viruses, or to a VSV-G-pseudotyped HIV-1 construct causing single-round HIV-1 infection. We observed that HLA-G^+^ CD4 Treg were significantly more susceptible to HIV-1 infection than autologous HLA-G^−^ CD4 T cells; this was true both for *in vitro* activated cells and for cells directly infected *ex-vivo* (Figure 6A–C, E). This enhanced susceptibility was in line with higher expression of the HIV-1 co-receptors, CXCR4 and CCR5, on HLA-G^+^ CD4 Treg, in comparison to HLA-G^−^ CD4 T cells (Figure 6D/F). These data suggest that reduction of circulating HLA-G^+^ CD4 Treg in progressive HIV-1 infection may, at least in part, be due to their enhanced susceptibility to HIV-1 infection.

## Discussion

Regulatory T lymphocytes can influence immune homeostasis by suppressing innate and adaptive effector cell activity, and in this way may importantly modulate immune defense mechanisms against HIV-1 [31]. The majority of currently available data indicate that classical CD25^hi^ CD127^lo^ Treg are expanded during chronic progressive HIV-1 infection [32], [33], [34], [35], [36], [37] and may worsen spontaneous HIV-1 disease progression by potently suppressing functional activities of HIV-1-specific T cell responses [5], [17], [38]. Here, we demonstrate several numerical and functional aspects of non-classical HLA-G-expressing Treg in HIV-1 infection that clearly distinguish them from these recognized characteristics of classical Treg. We found that absolute numbers and relative proportions of HLA-G-expressing Treg are diminished in progressive HIV-1 infection, that they are inversely correlated to phenotypic markers of immune activation, and that they may have a functional role for reducing bystander immune activation, while only minimally suppressing proliferative activities of HIV-1-specific T cells. In contrast, an alternative population of non-classical Treg expressing the TGF-β latency-associated antigen (LAP) was not correlated to immune activation during HIV-1 infection and weakly affected immune activation in functional assays. Overall, these data suggest that HLA-G-expressing Treg may contribute to balancing and fine-tuning anti-viral immune activity and bystander immune activation during HIV-1 infection.

HLA-G^+^ Treg represent a relatively recently discovered group of suppressive T cells that can inhibit the activation and proliferation of T cells after TCR triggering with CD3/CD28 antibodies. However, how HLA-G^+^ Treg functionally compare to classical Treg in terms of their ability to suppress virus-specific T cells or TCR-independent bystander activation of lymphocytes remained unclear. Our data show that HLA-G^+^ Treg do not effectively inhibit proliferation of HIV-1- and CMV-specific T cells, compared to the effects of classical Treg in HIV controllers. In contrast, we observed a seemingly stronger ability of HLA-G^+^ Treg to reduce TCR-independent bystander activation of T cells, using an assay that excludes TCR cross-reactivity as a possible source of activation in heterologous T cells. Yet, due to the numeric reduction of HLA-G^+^ Treg in progressive HIV-1 infection, all functional effects of these cells could not be evaluated using cells from this particular patient population. Whether functional properties of HLA-G^+^ Treg from HIV-1 progressors or HAART-treated patients resemble those of HIV-1 negative persons, or exhibit an altered or dysfunctional profile, remains to be investigated. Nevertheless, our results suggest that HLA-G^+^ Treg differ from alternative Treg populations by a unique profile of suppressive functions that may allow for reducing bystander immune activation while simultaneously minimizing inhibitory effects on virus-specific T cell immune responses. The preservation of this HLA-G-expressing Treg population in HIV-1 controllers may represent an additional immunological feature of this specific patient population.

This work demonstrates that in contrast to classical Treg, HLA-G-expressing Treg progressively decline during advanced HIV-1 infection. This selective loss of HLA-G^+^ Treg during advanced HIV-1 infection may, in conjunction with other mechanisms, contribute to immune overactivation during progressive HIV-1 infection. The reduction of HLA-G^+^ CD4 Treg during progressive HIV-1 infection may be related to their increased susceptibility to HIV-1 infection, which is likely due to enhanced expression of the viral co-receptors CCR5 and CXCR4 demonstrated in this study. An upregulation of these chemokine receptors may also lead to elevated sequestration of HLA-G^+^ Treg into inflamed tissues, where these cells were indeed preferentially observed in previous investigations [9], [39]. However, in our study, we did not find any positive evidence for a selective enrichment of HLA-G^+^ CD4 and CD8 Treg in lymphoid tissues, either in HAART-treated or in untreated HIV-1 patients; but this observation in a limited number of patients does not exclude the possibility of tissue compartmentalization of HLA-G^+^ Treg in HIV-1 infection. In addition, the specific reason for the loss of HLA-G^+^ CD8 Treg in untreated progressive HIV-1 infection remains unclear and warrants further investigation.

Over the recent years, HIV-1 infection has increasingly been recognized as a chronic inflammatory condition characterized by elevated T cell immune activation [40]. The mechanisms leading to this abnormal immune activation are most likely multifactorial and include direct stimulation of T cells by HIV-1 antigens, as well as direct TCR-mediated activation of T cells by alternative viral and bacterial antigens that challenge the host during conditions of HIV-1 associated immune deficiency. TCR-independent bystander immune activation does not seem to play a significant role under physiologic conditions, however, increasing data suggest that bystander activation represents a major driving factor for pathological immune activation during progressive HIV-1 infection. For instance, bystander activation occurs mainly through cytokines, including interferon-α/β, IL-2 and IL-15 [41], which are all increased in HIV-1 infection and represent independent and accurate predictors of disease progression [42]. Moreover, the majority of activated T cells in HIV-1 infected patients typically do not exhibit phenotypic markers of recent TCR stimulation [43], suggesting that their activation occurred by TCR-independent processes. In addition, activation of T cells specific for Influenza virus has been documented during HIV-1 infection in the absence of serological evidence of Influenza co-infection, or detectable TCR cross-reactivity between HIV-1 and Influenza antigens [44]. Interestingly, our data suggest that T cells activated by bystander mechanisms may have a higher susceptibility to inhibitory effects of HLA-G^+^ Treg, likely because they do not downregulate the HLA-G receptor LILRB1 in a similar way as T cells activated by TCR triggering. These observations indicate that TCR-dependent and TCR-independent mechanisms of immune activation are associated with altered susceptibilities to classical and non-classical Tregs, and shed new light on target cell characteristics that influence inhibitory effects of Tregs. By selectively reducing the deleterious effects of TCR-independent bystander activation, HLA-G^+^ Treg may provide a previously unrecognized form of immune protection against HIV-1 associated disease manifestations.

## Supporting Information

Figure S1 **Analysis of classical and non-classical Tregs in HIV-infected patients.** Representative dot plots reflect co-expression of HLA-G, CD25 and FoxP3 in patients with different rates of HIV-1 disease progression and in a healthy individual.
(TIFF)

Figure S2 **Characterization of classical Treg in HIV-1-infected persons with different rates of HIV-1 disease progression.** (A) Box and Whisker plots summarizing the proportions and absolute counts of CD25^hi^ CD127^lo^ CD4 Treg in indicated study cohorts. Significance was determined by ANOVA, followed by post-hoc analysis with Tukey's Multiple Comparison Test. (B) Correlation between proportions of CD25^hi^ CD127^lo^ CD4 Treg and levels of immune activation. Spearman's correlation coefficient is shown.
(TIFF)

Figure S3 **T cell subset distribution of HLA-G-expressing and bulk CD4 (A) and CD8 (B) T cells in indicated study cohorts.** Significance was tested by Mann Whitney U test between cohorts within HLA-G^+^ or bulk T cells, and by paired T test between HLA-G^+^ and corresponding bulk T cells.
(TIFF)

Figure S4 **Phenotypic analysis of HLA-G- and LAP-expressing Tregs in HIV-1 infected persons.** Surface expression of CD57 and PD-1 in HLA-G- (A) or LAP- (B) expressing CD4 and CD8 T cells in indicated study cohorts. Data from corresponding bulk T cell populations are indicated for reference purposes. Mann Whitney U test was used to analyze differences between study cohorts, and paired T test was used to compare paired HLA-G^+^ and corresponding bulk T cells.
(TIFF)

Figure S5 **Analysis of LAP^+^ Treg in HIV-1 patients.** (A): Representative dot plots reflecting the proportions of LAP^+^ CD4 and CD8 T cells in indicated study cohorts. FMO control reflects “fluorescence minus one” control without addition of LAP antibodies. (B): Box and Whisker plots summarizing the relative proportions and absolute numbers of LAP^+^ CD4 and CD8 T cells in indicated study cohorts. ANOVA followed by post-hoc analysis with Tukey's Multiple Comparison Test was used to determined significance. (C): Correlations between frequencies of LAP^+^ CD4 and CD8 T cells and total CD4 T cell counts in controllers (n = 16), progressors (n = 14) and HIV seronegative individuals (n = 7). (D): Correlations between proportions of LAP^+^ Treg and CD8 T cell immune activation determined by surface expression of CD38 and HLA-DR in controllers (n = 13), progressors (n = 7) and HIV seronegative individuals (n = 6) (D). (C/D): Spearman's correlation coefficient is shown.
(TIFF)

Figure S6 **T cell subset distribution of LAP-expressing and bulk CD4 (A) and CD8 (B) T cells in indicated study cohorts.** Mann Whitney U test was used to analyze differences between study cohorts, and paired T test was used to compare paired HLA-G^+^ and corresponding bulk T cells.
(TIFF)

Figure S7 **LAP^+^ Treg weakly inhibit proliferative activities of HIV-1-specific cytotoxic T cells.** (A): Representative dot plots reflecting proliferative activities of HIV-1-specific CD8 T cells from HIV controllers following incubation with indicated autologous Treg subsets or LAP^−^ CD25^−^ control cells. (B): Cumulative data from n = 6 study subjects reflecting the Treg-mediated suppression of HIV-1-specific CD8 T cell proliferation. Significance was tested by paired T test.
(TIFF)

Figure S8 **Non-classical Treg do not affect cytokine secretion properties of HIV-1-specific T cells.** Cumulative data indicating the proportion of IFN-γ^+^ (A) or IL-2^+^ (B) CD4 and CD8 T cells following exposure to indicated autologous Treg populations, or LAP^−^ HLA-G^−^ CD25^−^ control CD4 T cells in n = 5 HIV-1 controllers. Significance was tested by paired T test.
(TIFF)

Figure S9 **HLA-G-expression in cells and in the culture supernatant.** (A) Western blots reflecting cell-associated HLA-G in isolated HLA-G^+^ and HLA-G^−^ T cell subsets, and in culture supernatants from these two different cell populations. (B): Quantitative assessment of cell-associated and soluble HLA-G protein from HLA-G^+^ and HLA-G^−^ T cells from n = 4 HIV-1 negative subjects. Significance was tested by paired T test.
(TIFF)
