# Supplementary figures and images for: Functional Characterization of HLA-G+ Regulatory T Cells in HIV-1 Infection
Source: PLoS Pathog. 2013 Jan 31;9(1):e1003140. doi: 10.1371/journal.ppat.1003140 (PMC3561210; doi:10.1371/journal.ppat.1003140)

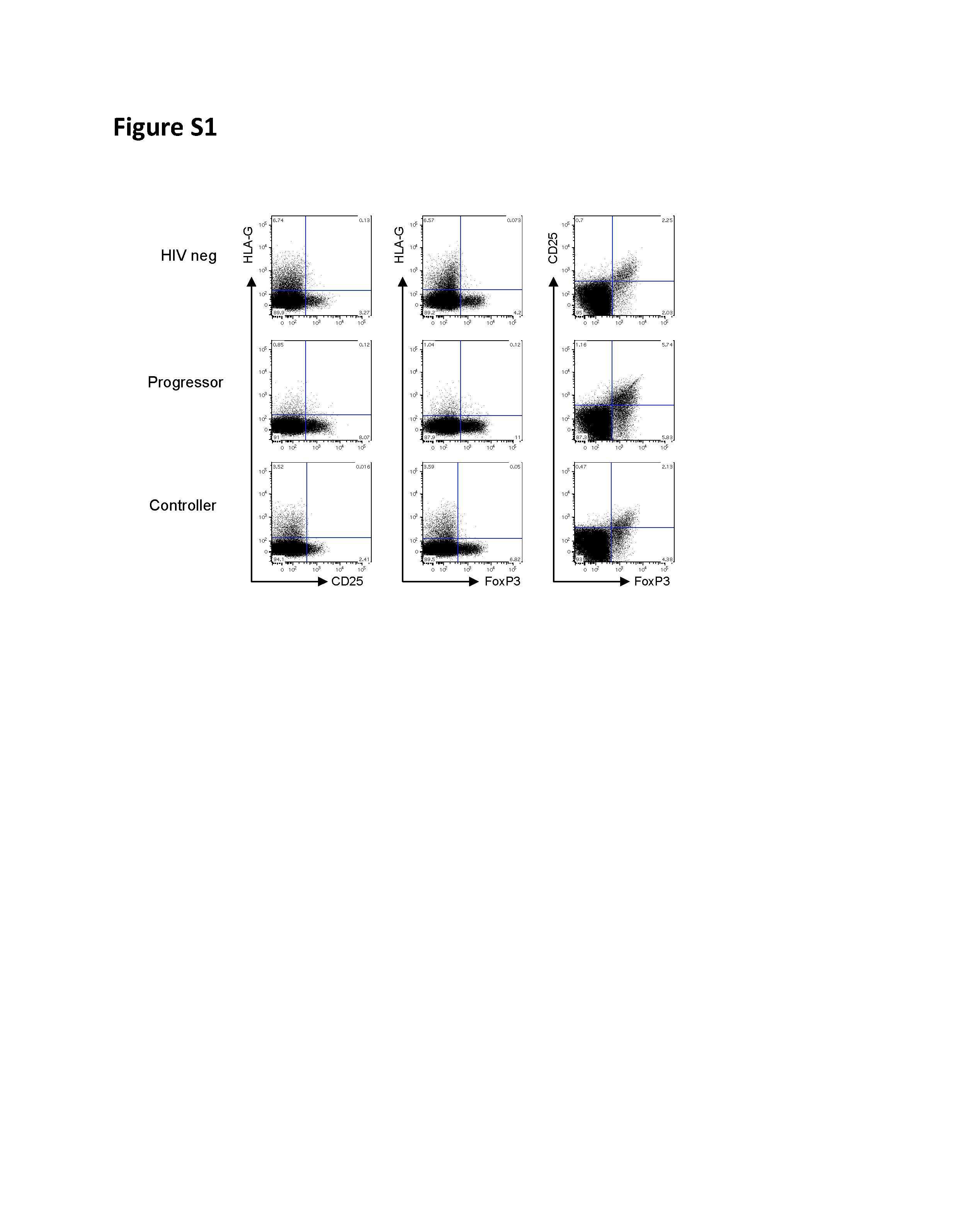

Supplement: Figure S1 — Analysis of classical and non-classical Tregs in HIV-infected patients. Representative dot plots reflect co-expression of HLA-G, CD25 and FoxP3 in patients with different rates of HIV-1 disease progression and in a healthy individual. (TIFF) [file ppat.1003140.s001.tiff]

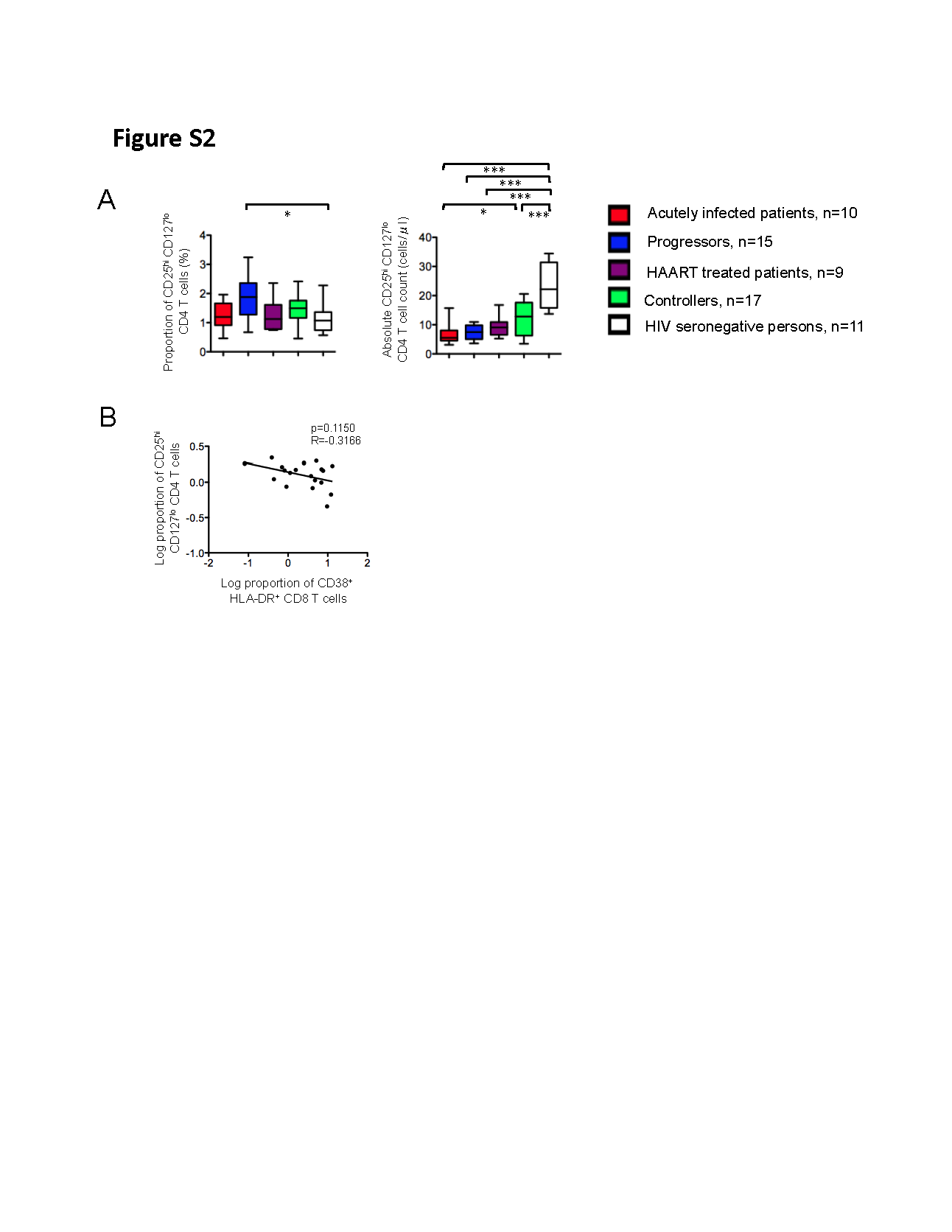

Supplement: Figure S2 — Characterization of classical Treg in HIV-1-infected persons with different rates of HIV-1 disease progression. (A) Box and Whisker plots summarizing the proportions and absolute counts of CD25hi CD127lo CD4 Treg in indicated study cohorts. Significance was determined by ANOVA, followed by post-hoc analysis with Tukey's Multiple Comparison Test. (B) Correlation between proportions of CD25hi CD127lo CD4 Treg and levels of immune activation. Spearman's correlation coefficient is shown. (TIFF) [file ppat.1003140.s002.tiff]

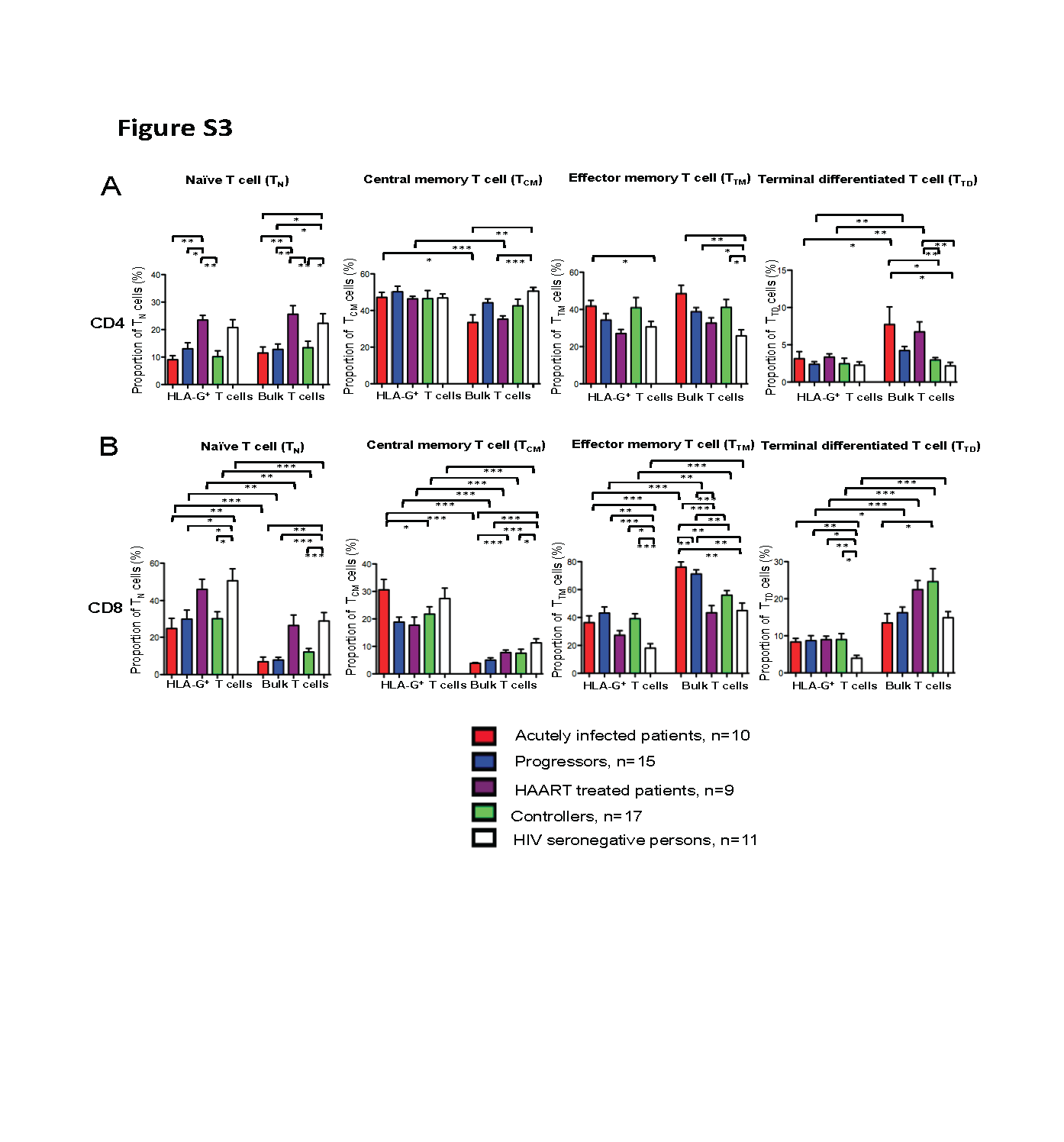

Supplement: Figure S3 — T cell subset distribution of HLA-G-expressing and bulk CD4 (A) and CD8 (B) T cells in indicated study cohorts. Significance was tested by Mann Whitney U test between cohorts within HLA-G+ or bulk T cells, and by paired T test between HLA-G+ and corresponding bulk T cells. (TIFF) [file ppat.1003140.s003.tiff]

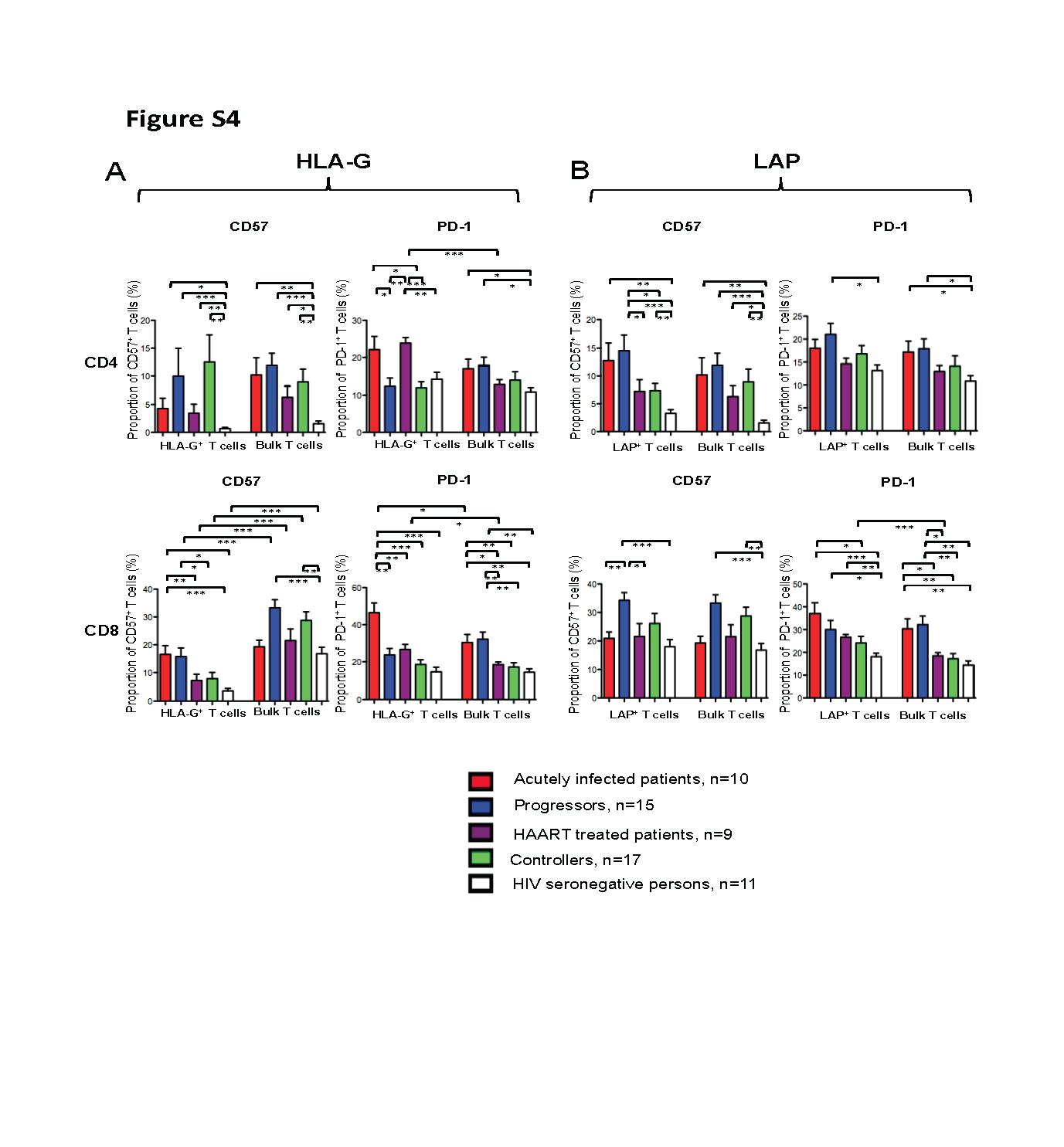

Supplement: Figure S4 — Phenotypic analysis of HLA-G- and LAP-expressing Tregs in HIV-1 infected persons. Surface expression of CD57 and PD-1 in HLA-G- (A) or LAP- (B) expressing CD4 and CD8 T cells in indicated study cohorts. Data from corresponding bulk T cell populations are indicated for reference purposes. Mann Whitney U test was used to analyze differences between study cohorts, and paired T test was used to compare paired HLA-G+ and corresponding bulk T cells. (TIFF) [file ppat.1003140.s004.tiff]

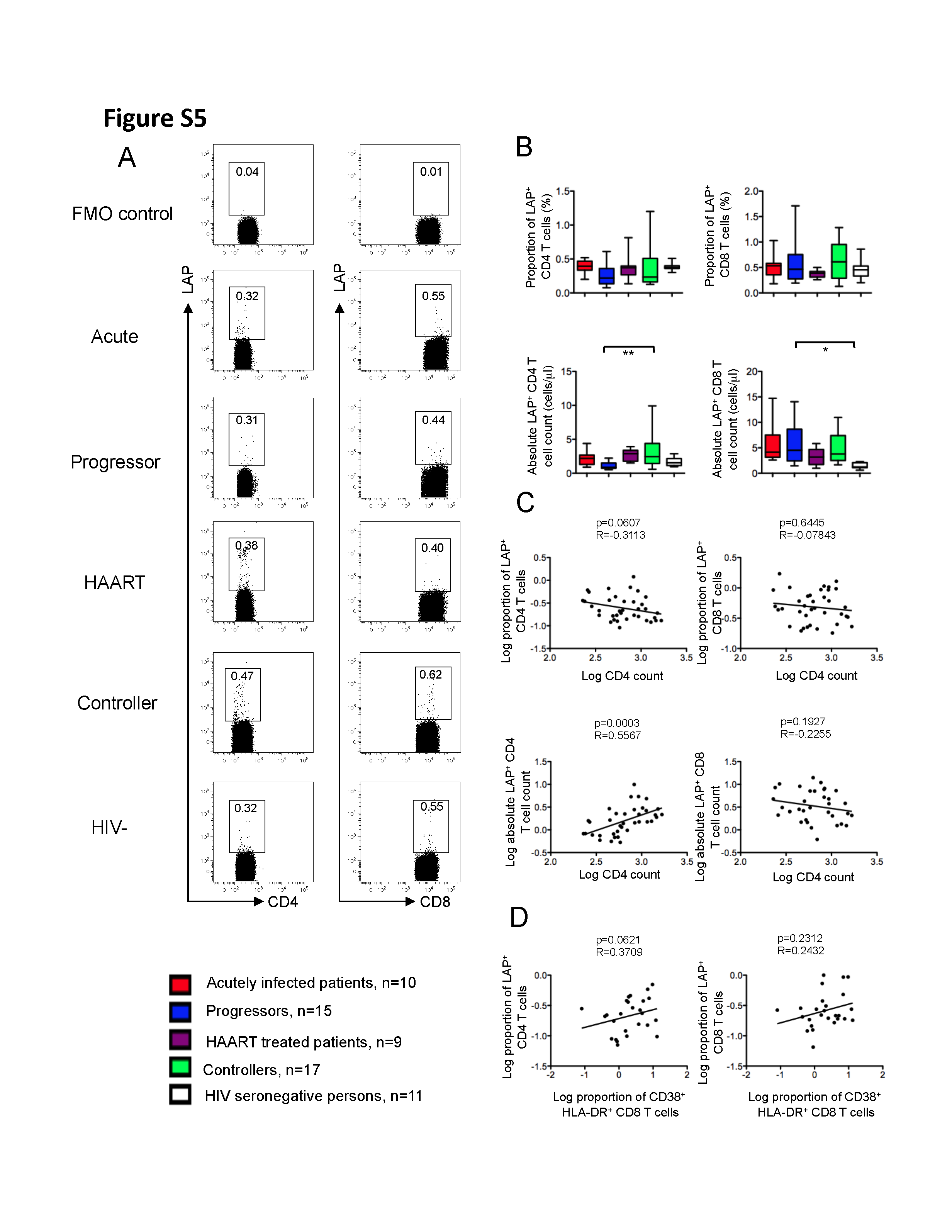

Supplement: Figure S5 — Analysis of LAP+ Treg in HIV-1 patients. (A): Representative dot plots reflecting the proportions of LAP+ CD4 and CD8 T cells in indicated study cohorts. FMO control reflects “fluorescence minus one” control without addition of LAP antibodies. (B): Box and Whisker plots summarizing the relative proportions and absolute numbers of LAP+ CD4 and CD8 T cells in indicated study cohorts. ANOVA followed by post-hoc analysis with Tukey's Multiple Comparison Test was used to determined significance. (C): Correlations between frequencies of LAP+ CD4 and CD8 T cells and total CD4 T cell counts in controllers (n = 16), progressors (n = 14) and HIV seronegative individuals (n = 7). (D): Correlations between proportions of LAP+ Treg and CD8 T cell immune activation determined by surface expression of CD38 and HLA-DR in controllers (n = 13), progressors (n = 7) and HIV seronegative individuals (n = 6) (D). (C/D): Spearman's correlation coefficient is shown. (TIFF) [file ppat.1003140.s005.tiff]

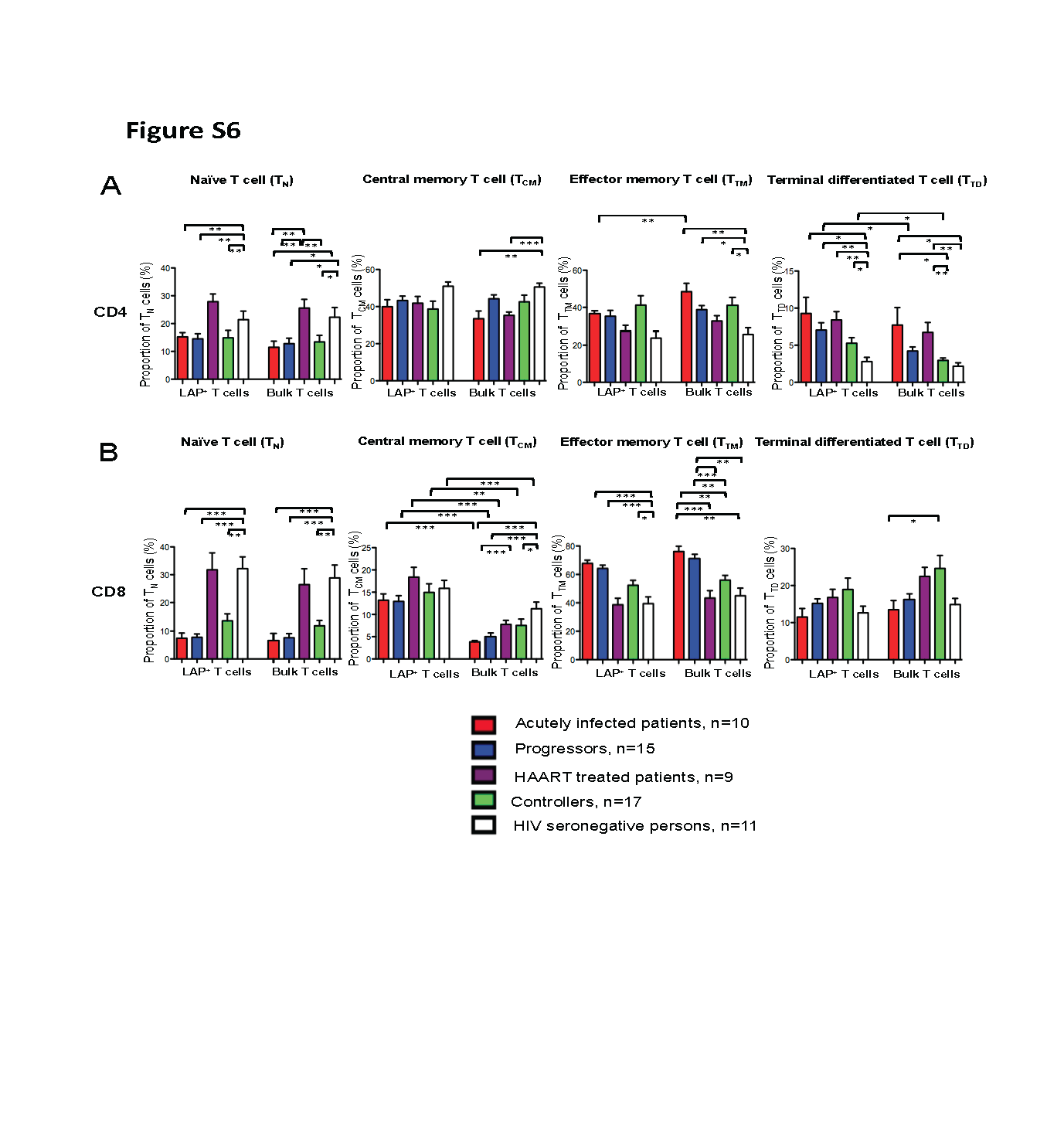

Supplement: Figure S6 — T cell subset distribution of LAP-expressing and bulk CD4 (A) and CD8 (B) T cells in indicated study cohorts. Mann Whitney U test was used to analyze differences between study cohorts, and paired T test was used to compare paired HLA-G+ and corresponding bulk T cells. (TIFF) [file ppat.1003140.s006.tiff]

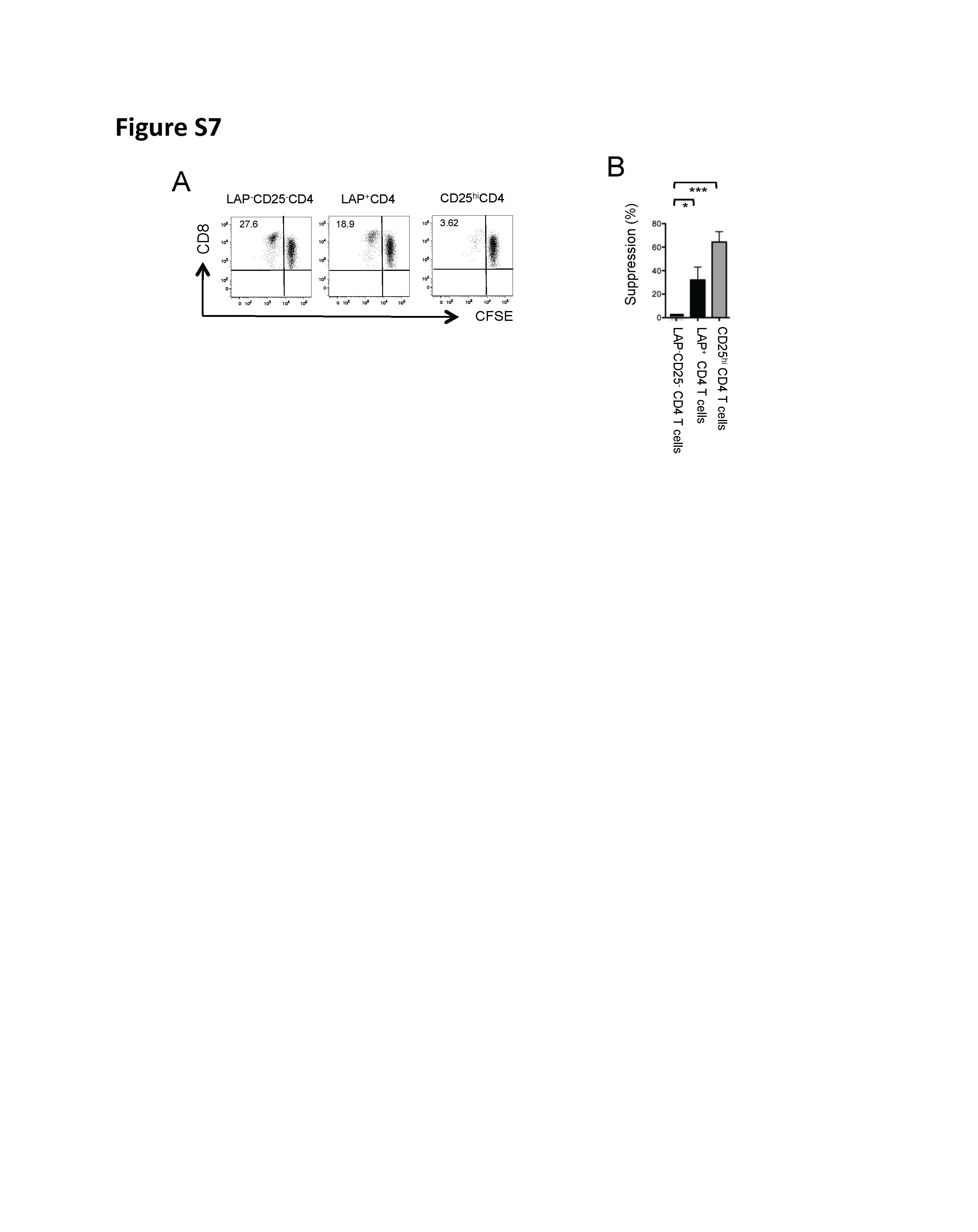

Supplement: Figure S7 — LAP+ Treg weakly inhibit proliferative activities of HIV-1-specific cytotoxic T cells. (A): Representative dot plots reflecting proliferative activities of HIV-1-specific CD8 T cells from HIV controllers following incubation with indicated autologous Treg subsets or LAP− CD25− control cells. (B): Cumulative data from n = 6 study subjects reflecting the Treg-mediated suppression of HIV-1-specific CD8 T cell proliferation. Significance was tested by paired T test. (TIFF) [file ppat.1003140.s007.tiff]

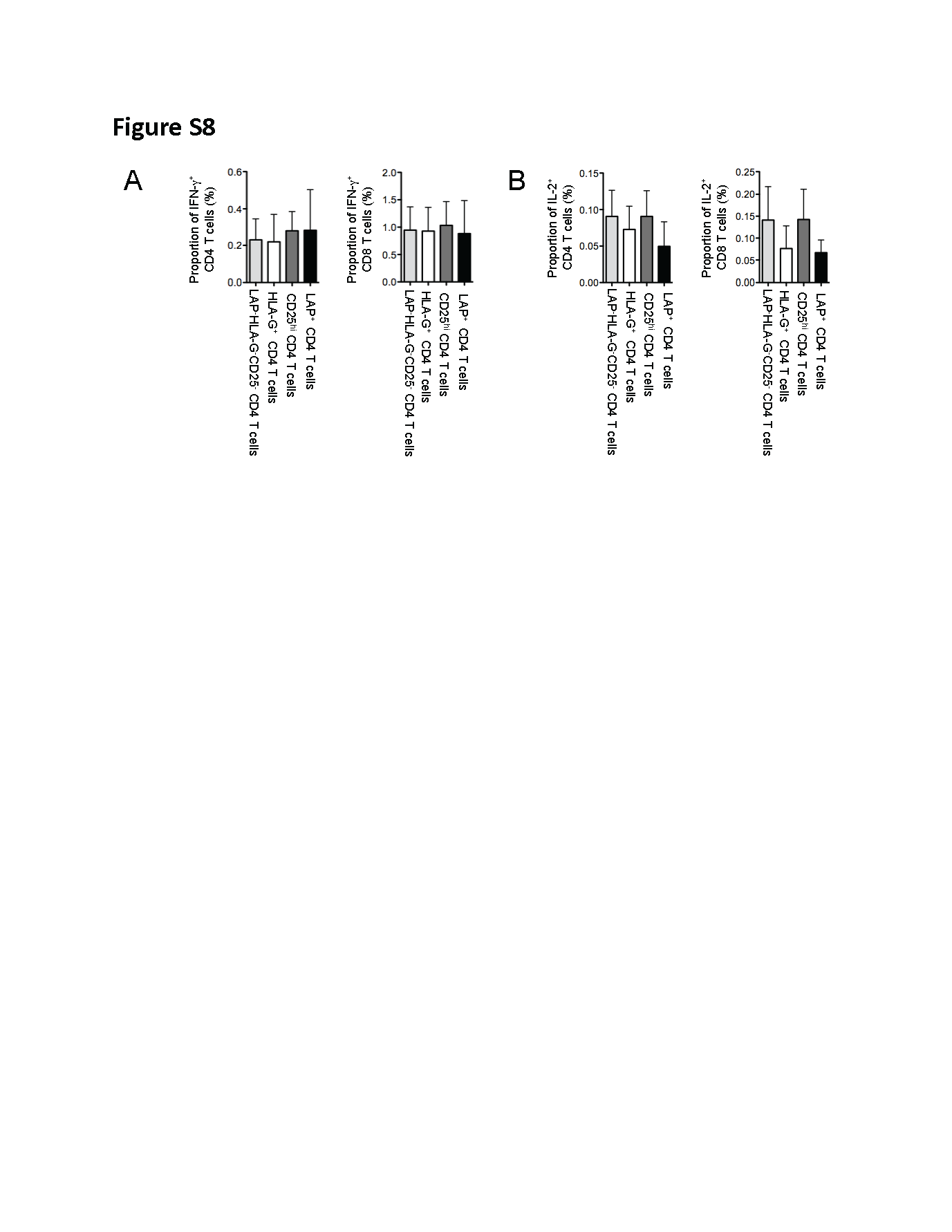

Supplement: Figure S8 — Non-classical Treg do not affect cytokine secretion properties of HIV-1-specific T cells. Cumulative data indicating the proportion of IFN-γ+ (A) or IL-2+ (B) CD4 and CD8 T cells following exposure to indicated autologous Treg populations, or LAP− HLA-G− CD25− control CD4 T cells in n = 5 HIV-1 controllers. Significance was tested by paired T test. (TIFF) [file ppat.1003140.s008.tiff]

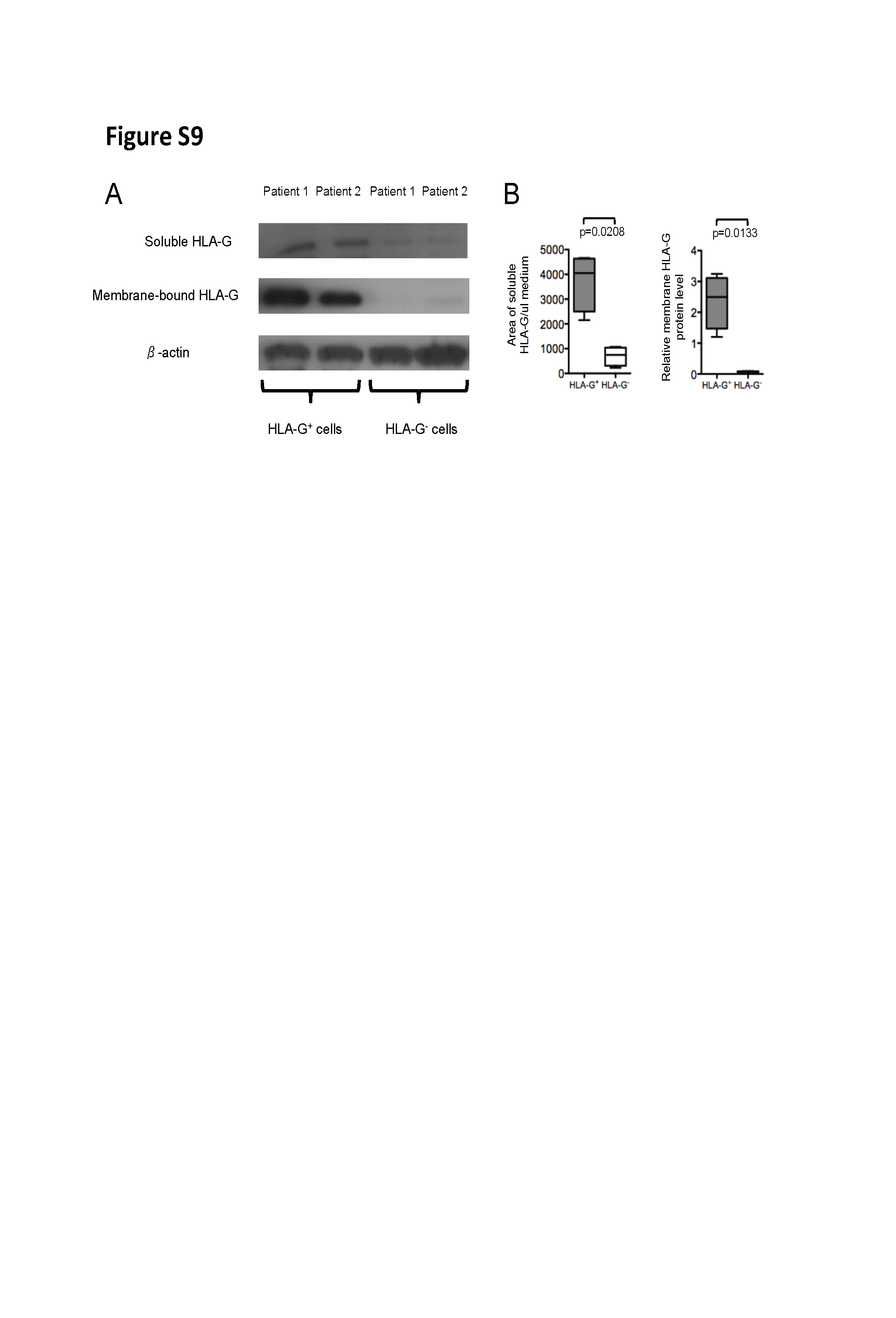

Supplement: Figure S9 — HLA-G-expression in cells and in the culture supernatant. (A) Western blots reflecting cell-associated HLA-G in isolated HLA-G+ and HLA-G− T cell subsets, and in culture supernatants from these two different cell populations. (B): Quantitative assessment of cell-associated and soluble HLA-G protein from HLA-G+ and HLA-G− T cells from n = 4 HIV-1 negative subjects. Significance was tested by paired T test. (TIFF) [file ppat.1003140.s009.tiff]
